# American Medical Association Officers

**Published:** 1905-08

**Authors:** 


					﻿American Medical Association Officers.—President, Df.
William J. Mayo, Rochester, Minn.; fiirst vice president, Brigadier
General Walter Wyman, Washington, D. C.; second vice president,
Dr. J. K. McKenzie, Portland; third vice president/Dr. E. S.
Talbot, Chicago; fourth vice president, Dr. Edwin D. Martin, New
Orleans; general secretary, Dr. George H. Simmons, Chicago;
treasurer, Dr. Frank Billings, Chicago; member board of direc-
tors, Dr. E. E. Montgomery, Pennsylvania. Meet at Boston next
year.
				

